# Longitudinal Evaluation of Glucose Profile and Obesity Using Continuous Glucose Monitoring, Bioelectrical Impedance Analysis, and Computed Tomography Fat Scan in a Patient Who Achieved Diabetes Remission After Laparoscopic Sleeve Gastrectomy Duodenojejunal Bypass

**DOI:** 10.1016/j.aed.2025.11.007

**Published:** 2025-11-26

**Authors:** Yoshinori Ozeki, Takayuki Masaki, Takaaki Noguchi, Shotaro Miyamoto, Yuichi Yoshida, Koro Gotoh, Yuichi Endo, Masafumi Inomata, Hirotaka Shibata

**Affiliations:** 1 Department of Endocrinology, Metabolism, Rheumatology and Nephrology, Faculty of Medicine, Oita University, Oita, Japan; 2 Obesity and Diabetes Center for Advanced Medicine, Faculty of Medicine, Oita University, Oita, Japan; 3 Department of Practical Nursing Sciences, Faculty of Medicine, Oita University, Oita, Japan; 4 Faculty of Welfare and Health Sciences, Oita University, Oita, Japan; 5 Department of Gastroenterological and Pediatric Surgery, Faculty of Medicine, Oita University, Oita, Japan

**Keywords:** Obesity Management, Metabolic surgery, Body Composition, Abdominal Fat, Glucose variability

## Abstract

**Background/Objective:**

Laparoscopic sleeve gastrectomy/duodenojejunal bypass (LSG/DJB) aids weight loss and improves type 2 diabetes mellitus (T2DM) in patients with obesity. Herein, we present a 1-year longitudinal evaluation using continuous glucose monitoring (CGM), bioelectrical impedance analysis (BIA), and computed tomography (CT) fat scans after LSG/DJB in a patient with obesity and T2DM.

**Case Presentation:**

Our patient was a 33-year-old female with obesity and T2DM who had been treated with intensive insulin therapy (50 units/d) before LSG/DJB. Preoperatively, the patient had uncontrolled diabetes, with a fasting blood glucose level of 252 mg/dL and HbA1c of 10.0%. Moreover, the CGM showed 1%, 99%, and 0% time-in range (TIR), time-above range, and time-below range, respectively. LSG/DJB significantly increased the 3-, 6-, and 12-month TIR to 96%, 93%, and 89%, respectively, and decreased the time-above range to 4%, 7%, and 11%, respectively, indicating high-dose insulin withdrawal and complete diabetes remission. Twelve months post-LSG/DJB, effective abdominal fat mass loss was observed on CT. Furthermore, both FM and the percentage of fat mass were reduced, and muscle mass was maintained according to the BIA.

**Discussion:**

Effective weight loss, abdominal FM loss, and complete diabetes remission were observed after LSG/DJB, according to BIA and CT. The significant increase in TIR suggests that this therapeutic approach may improve the prognosis of patients with T2DM and obesity.

**Conclusion:**

CGM, BIA, and CT fat scans are highly useful for the long-term management of obesity and diabetes after LSG/DJB surgery.

**Highlights**

• Laparoscopic sleeve gastrectomy/duodenojejunal bypass is a promising bariatric surgical procedure for obesity and type 2 diabetes mellitus
• Postoperative anemia can make A1c level a poor indicator for type 2 diabetes mellitus
• Post-bariatric surgery obesity assessment should evaluate visceral fat and muscle mass
• CGM, BIA, and fat scans are useful for long-term obesity and diabetes management post-surgery

**Clinical Relevance**

This is a case of an obese diabetic patient with an A1C level of over 10%. The patient required high-dose insulin injections and had poor insulin secretory capacity but was able to wean off insulin therapy within a few months and achieve complete remission of diabetes. We assessed body composition and glucose variability using bioelectrical impedance analysis/computed tomography and continuous glucose monitoring after laparoscopic sleeve gastrectomy/duodenojejunal bypass for the first time. Continuous glucose monitoring, bioelectrical impedance analysis methods, and fat scans are highly useful for managing obesity and diabetes long-term after surgery in patients undergoing laparoscopic sleeve gastrectomy/duodenojejunal bypass procedures.

## Introduction

Bariatric and metabolic surgery is effective for obesity and type 2 diabetes mellitus (T2DM), with a high rate of improvement and, in many cases, remission (normal hemoglobin A1c (HbA1c) levels without the use of medication).^1^ Laparoscopic sleeve gastrectomy (LSG) is the most commonly performed weight loss and diabetes improvement surgery worldwide; however, gastrointestinal bypass is more effective for diabetes remission and low density lipoprotein (LDL)-cholesterol concentrations than LSG.^2^ Several types of surgery involve gastrointestinal bypass; however, in Japan, owing to the high incidence of stomach cancer, LSG/duodenojejunal bypass (LSG/DJB), which enables postoperative observation of the stomach, is mainly performed for the treatment of obesity and T2DM.^3^^,^^4^

A meta-analysis showed that continuous glucose monitoring (CGM) is associated with a high reduction in glucose and HbA1c levels, increased time-in range (TIR), and decreased time-above range (TAR).^5^ CGM also offers a more accurate assessment of hypoglycemic episodes than traditional blood glucose testing methods, making it useful for managing blood glucose levels, including in the context of bariatric surgery.^6^ However, most CGM analyses are findings of LSG or Roux-en-Y bypass surgery, and no long-term longitudinal studies have examined LSG/DJB to date. In addition, most studies have used CGM for 3–7 days for investigating postoperative hypoglycemia and dumping syndrome.^7^^,^^8^ Moreover, the evaluation of obesity should be based not only on weight but also on a longitudinal evaluation of body composition and visceral fat, for which bioelectrical impedance analysis (BIA) and computed tomography (CT) are useful. This study aimed to evaluate body composition and metabolic profiles using CGM, BIA, and CT fat scans after LSG/DJB in a patient with obesity and T2DM.

## Case Presentation

A 33-year-old female with obesity and T2DM was initiated on insulin treatment after being admitted to a nearby hospital for educational purposes. As she wanted to have children, she continued to receive intensive insulin therapy after discharge. She started taking insulin aspart in the morning, 6 units in the afternoon, and 6 units in the evening. The patient was discharged from the hospital and continued insulin therapy; however, the glycemic control was poor owing to obesity. She gradually gained weight; therefore, her previous hospital introduced glucagon-like peptide-1 (GLP-1) receptor agonists and sodium-glucose cotransporter-2 (SGLT2) inhibitors. However, the GLP-1 receptor agonist was discontinued owing to its high cost and side effects. Her complications included dyslipidemia, a family history of diabetes in her mother, and heart disease in her father. She was referred to our hospital because she wanted to undergo LSG/DJB for T2DM and obesity.

Physical examination revealed that the patient weighed 77.0 kg and had a body mass index (BMI) of 30.2 kg/m^2^ (Table 1). Blood tests showed the following: fasting plasma glucose (FPG), 235 mg/dL (13.1 mmol/L; reference range, 70-109 mg/dL [3.9-6.0 mmol/L]); HbA1c, 10.1% (74.6 mmol/mol; reference range, 4.6% to 5.6% [33.9-41.4 mmol/mol]); triglyceride, 228 mg/dL (2.6 mmol/L; reference range, 50-150 mg/dL [0.6-1.7 mmol/L]); aspartate aminotransferase (AST), 65.1 IU/L (reference range, 11-24 IU/L); alanine aminotransferase (ALT), 128.1 IU/L (reference range, 11-24 IU/L); glutamic pyruvic transaminase, 78.3 IU/L (reference range, 11-24 IU/L); and normal hypothalamic pituitary–adrenal axis and thyroid results. The fasting serum C-peptide level was 1.6 ng/mL (reference range, 0.6-2.1 ng/mL), and the C-peptide index was 0.68.

**Table 1:** Body Weight, Plasma Metabolic Parameters, and Body Composition Pre- and Post-LSG/DJB

	pre-LSG/DJB	3 mo	6 mo	9 mo	12 mo
Body weight (kg)	77.0	64.2	57.1	52.7	50.3
%TBWL	0.0	16.6	25.8	31.6	34.7
BMI (kg/m^2^)	30.2	25.1	22.3	20.6	19.7
Fasting plasma glucose (mg/dL)	235	88	93	93	90
HbA1c (%)	10.0	5.4	5.1	5.3	5.3
Serum C-peptide (ng/mL)	1.6				1.5
AST (IU/L)	65.1	21.5	14.1	16.8	28.1
ALT (IU/L)	128.1	21.7	11.9	15.4	30.7
GTP (IU/L)	78.3	15.8	11.3	14.1	14.0
Creatinine (mg/dL)	0.42	0.50	0.54	0.58	0.47
FM (%)	45.3	34.0	26.0	21.7	18.7
FM (kg)	34.9	21.8	14.8	11.4	9.4
Total MM (kg)	39.8	39.9	39.8	38.8	38.5
Skeletal MM (kg)	23.1	22.6	22.4	21.8	21.8
Skeletal MM/BW	0.30	0.35	0.39	0.41	0.43

Abbreviations: ALT = alanine aminotransferase; AST = aspartate aminotransferase; BMI = body mass index; BW = body weight; FM = fat mass; GTP = glutamic pyruvic transaminase; HbA1c = hemoglobin A1c; LSG/DJB = laparoscopic sleeve gastrectomy/duodenojejunal bypass; MM = muscle mass; TBWL = total body weight loss.

Body fat mass and fat mass percentage (% FM), assessed in the fasting state with normal fluid intake using BIA (InBody 770; InBody Japan, Ltd., Tokyo, Japan), were 34.9 kg and 45.3%, respectively. Blood and urine tests were performed to detect dehydration and overhydration. The body skeletal muscle mass and muscle percentage (% MM) were 23.1 kg and 30.0%, respectively (Table 1). Preoperatively, the subcutaneous adipose tissue (SAT) and visceral adipose tissue (VAT) areas at the level of the umbilicus were 200.4 and 116.3 cm^2^, respectively, according to a CT fat scan software (*N*2 Systems, Osaka, Japan) (Table 2).

**Table 2:** Subcutaneous Adipose Tissue Area and Visceral Adipose Tissue Area at the Umbilicus

	pre-LSG/DJB	12 mo
Subcutaneous adipose tissue area (cm^2^)	200.4	78.9
Visceral adipose tissue area (cm^2^)	116.3	30.5

Abbreviation: LSG/DJB = laparoscopic sleeve gastrectomy/duodenojejunal bypass.

A Freestyle Libre Pro continuous glucose monitor (CGM; Abbott Diabetes Care, Tokyo, Japan) was used to assess the glucose variability. Using CGM, we obtained data on daily glucose levels: TIR, defined as the percentage of time with a glucose level of 70 to 180 mg/dL; TBR, defined as the percentage of time with a glucose level of <70 mg/dL; and TAR, defined as the percentage of time with a glucose level >180 mg/dL. CGM displayed 1%, 99%, and 0% TIR, TAR, and TBR, respectively (Fig.). The patient was diagnosed with obesity, hyperlipidemia, and hyperglycemia, with decreased insulin secretion.

**Fig:**
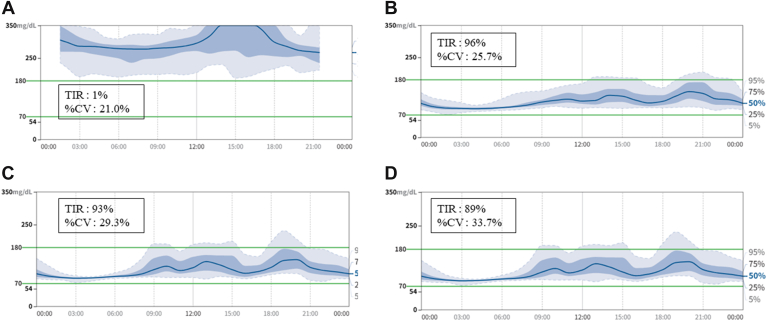
Mean glucose level according to the CGM pre-versus post-LSG/DJB: (A) Pre-LSG/DJB; (B) At 3 months post-LSG/DJB; (C) At 6 months post-LSG/DJB; (D) At 12 months post-LSG/DJB. % CV = coefficient of variation (%); CGM = continuous glucose monitor; LSG/DJB = laparoscopic sleeve gastrectomy/duodenojejunal bypass; TIR = time in range.

In March 2024, the patient underwent LSG/DJB for obesity and diabetes mellitus. Prediction scores that combine several factors may predict postoperative improvements in patients with diabetes. Representative scores include the ABCD and DiaRem, which can effectively predict improvements. The patient’s pre-LSG/DJB ABCD and DiaRem scores were 3 and 19, respectively. The following were prescribed: dapagliflozin 5 mg/d; rosuvastatin 2.5 mg/d; insulin detemir 10 units just before breakfast; and insulin aspart 12 units in the morning, 14 units at lunch, and 14 units in the evening just before dinner. The LSG/DJB procedure adds an absorption-restriction effect by adding bypass surgery to LSG, which is a feeding-restriction surgery. Post-LSG, the duodenal bulb was resected, and the jejunum was anastomosed in a Roux-en-Y configuration to create a bypass. Digestion and absorption were suppressed by setting the biliopancreatic limb from the ligament of Treitz to the jejunojejunostomy to approximately 100 to 150 cm and the nutritional limb to the duodenojejunostomy to approximately 100 cm. This procedure makes examining the stomach postoperatively easier and is considered more suitable than laparoscopic Roux-en-Y gastric bypass for use in Japan, which has a high incidence of gastric cancer.

Table 1 shows the time-course changes in body weight, BMI, body composition, and glucose levels post-LSG/DJB. Body weight and BMI decreased at 3, 6, 9, and 12 months post-LSG/DJB. At 3, 6, and 12 months post-LSG/DJB, HbA1c levels significantly improved, whereas FPG levels significantly improved (Table 1). Consequently, all antidiabetic medications, including insulin injections, were discontinued 6 months post-LSG/DJB. At 12 months post-LSG/DJB, the patient's fasting serum C-peptide level was 1.5 ng/mL, and the C-peptide index was 1.7. The AST, ALT, and triglyceride levels decreased post-LSG. Table 1 shows the changes in body composition after LSG/DJB. Both FM and % FM reduced and maintained post-LSG/DJB compared to those pre-LSG/DJB. MM and % MM were maintained at 3, 6, 9, and 12 months post-LSG/DJB (Table 1). Moreover, SAT and VAT at the level of the umbilicus were 78.9 and 30.5 cm^2^, respectively, 12 months post- LSG/DJB (Table 2).

Diabetes remission is defined as complete remission with the abolition of diabetes medication and an HbA1c level <6.0%. In this case, complete diabetes remission was observed pre-LSG/DJB and at 3, 6, 9, and 12 months post-LSG/DJB. Three months post-LSG/DJB, the mean interstitial glucose level was 118 mg/dL with a TBR of 0% (coefficient of variation [CV], 25.7%; Fig.). Six months post-LSG/DJB, the mean glucose level was 115 mg/dL with a TBR of 0% (CV, 29.3%). At 12 months post-LSG/DJB, the mean glucose level was 120 mg/dL with a TBR of 0% (CV, 33.7%). Similarly, TIR increased from 1% pre-LSG/DJB to 96%, 93%, and 89%, while TAR significantly decreased from 99% to 4%, 7%, and 11% at 3, 6, and 12 months post-LSG/DJB, respectively (Fig.). Both dietary adherence and nutritional status of the patients were good throughout the follow-up period. In addition, the patient exhibited no symptoms of dumping syndrome or hypoglycemia, as all TBR values were 0% (Fig.).

## Discussion

Randomized controlled trials of medical and surgical treatments for obesity-related T2DM have also been conducted, and the usefulness of surgical treatment has been reported in terms of improvements in both HbA1c and BMI.^9^ However, residual pancreatic β-cell function is essential for remission, and a predictive scoring system for T2DM remission has been published.^10^^,^^11^

A previous study described key predictors of T2DM remission, including low HbA1c and high C-peptide levels; high insulin doses have been associated with low remission rates after bariatric surgery.^10^ In this rare case, a patient with obesity, diabetes, and A1C >10% who required high-dose insulin injections and had poor insulin secretory capacity was weaned off insulin within a few months and achieved complete diabetes remission.

Although few cases of the effectiveness of LSG/DJB have been reported, it is a potentially promising bariatric surgical procedure for obesity and T2DM.^4^ This is the first study to evaluate the usefulness of CGM and BIA/CT for evaluating glucose profiles and obesity post-LSG/DJB. Effective weight loss, abdominal FM loss, and complete diabetes remission were observed post-LSG/DJB, according to BIA and CT fat scans. In addition, although the patient lost >20 kg post-LSG/DJB, almost no decrease in muscle mass was observed on the BIA. We have previously demonstrated that both BIA and CT fat scans are useful for evaluating body composition and visceral fat.^12^^,^^13^ We also noted a relationship between T2DM improvement and muscle maintenance,^12^ indicating that muscle maintenance is involved in diabetes remission.

Bariatric surgery is designed to help patients with severe obesity lose weight and boost their metabolisms. Significant efforts have been made to improve the management of T2DM.^8^^,^^9^ Over the past few years, improvements in CGM technology have improved pre- and post-surgical blood glucose management.^7^^,^^14^, ^15^, ^16^ High glycemic variability is frequently observed in individuals who undergo gastric bypass surgery.

A previous investigation detailed that the mean amplitude of glycemic excursions in patients who underwent gastric bypass surgery and patients with diabetes were 86 ± 58 and 66 ± 24 mg/dL, respectively,^14^ in contrast to those reported in a multi-ethnic study for the Hispanic population (21.6 ± 12.6 mg/dL). CGM has also proven useful for verifying the occurrence of hypoglycemia and dumping syndrome in individuals undergoing bariatric surgery.^15^ The primary applications of CGM are the identification of glucose patterns and the evaluation of the effectiveness of glycemic treatments. Most postoperative CGM analyses are based on data from LSG or Roux-en-Y bypass surgery, and long-term longitudinal data from LSG/DBJ studies are lacking. In addition, most studies have employed CGM for only a few days, focusing on postoperative hypoglycemia and dumping syndrome.^7^

In this case, the CGM showed 1%, 99%, and 0% TIR, TAR, and TBR, respectively. TIR increased significantly, and TAR decreased post-LSG/DJB. In addition, CGM showed good blood glucose fluctuations (25% to 35%) at 3, 6, and 12 months post-LSG/DJB and a TIR of approximately 90%.

Recently, TIR has been strongly associated with cardiovascular and diabetic complications. The significant increase in TIR and decrease in TAR observed with combination therapy using LSG/DJB surgery suggest that this therapeutic approach may improve the prognosis of patients with T2DM and obesity. Lower TAR and TIR (>70%) decrease the risk of microvascular and macrovascular damage in patients with diabetes.^17^ In addition, cardiovascular management is effective in reducing the risks of renal and cardiovascular events.^18^ This patient maintained a TIR of over 90% after LSG/DJB surgery, which may have contributed to improving the risk of diabetic complications. Although the mechanism underlying diabetes improvement by LSG/DJB has not been fully elucidated, it is possible that bariatric surgery improves obesity and diabetes through restriction, malabsorption, and hormonal modulation (such as increased GLP-1 secretion).^19^

Furthermore, our patient did not exhibit symptoms of dumping syndrome or hypoglycemia during CGM post-LSG/DJB. Providing regular dietary advice, including on carbohydrate intake and post-LSG/DJB surgery, may be helpful. This is the first case report of a 1-year longitudinal evaluation of the post-LSG/DJB glucose profile using a CGM.

This study had some limitations. First, because CGM measures glucose levels in the interstitial fluid, there may be a slight delay in values compared to the actual blood glucose levels. Second, the Freestyle Libre Pro reportedly produces lower glucose values than the actual measured values, a discrepancy that is more pronounced during low blood glucose periods, such as late at night or early in the morning.^18^ Third, BIA is simple, fast, and allows for repeated measurements; however, its limitations include errors in patients with cancer and body fluid imbalance and in assessing specific body composition. In contrast, CT is not affected by fluid status and effectively assesses visceral fat; however, its limitations include radiation exposure, making it unsuitable for frequent use.

In conclusion, our findings demonstrate that CGM, BIA, and CT fat scans are highly useful for the long-term management of obesity and T2DM post-LSG/DJB. In particular, detailed monitoring of blood glucose levels using CGM facilitates the management of hypoglycemia risk and the evaluation of diabetes remission post-LSG/DJB. The clinical application of CGM with LSG/DJB surgery is expected to improve future metabolic outcomes.

## Patient Consent

Informed consent has been obtained from the patient and included in this manuscript, in accordance with Elsevier’s guidelines for Patient Consent.

## Conflict of Interest

The authors have no conflicts of interest to disclose.

## Funding Statement

This study was supported by the 10.13039/501100001691JSPS 10.13039/501100001691KAKENHI (JP 21K16425). Sonal relationships which may be considered as potential competing interests.
